# Biological Activities of Secretory RNases: Focus on Their Oligomerization to Design Antitumor Drugs

**DOI:** 10.3389/fimmu.2019.02626

**Published:** 2019-11-26

**Authors:** Giovanni Gotte, Marta Menegazzi

**Affiliations:** Biological Chemistry Section, Department of Neuroscience, Biomedicine and Movement Sciences, University of Verona, Verona, Italy

**Keywords:** ribonucleases, RNase oligomers, domain swapping, cytotoxicity, antitumor activity

## Abstract

Ribonucleases (RNases) are a large number of enzymes gathered into different bacterial or eukaryotic superfamilies. Bovine pancreatic RNase A, bovine seminal BS-RNase, human pancreatic RNase 1, angiogenin (RNase 5), and amphibian onconase belong to the pancreatic type superfamily, while binase and barnase are in the bacterial RNase N1/T1 family. In physiological conditions, most RNases secreted in the extracellular space counteract the undesired effects of extracellular RNAs and become protective against infections. Instead, if they enter the cell, RNases can digest intracellular RNAs, becoming cytotoxic and having advantageous effects against malignant cells. Their biological activities have been investigated either *in vitro*, toward a number of different cancer cell lines, or in some cases *in vivo* to test their potential therapeutic use. However, immunogenicity or other undesired effects have sometimes been associated with their action. Nevertheless, the use of RNases in therapy remains an appealing strategy against some still incurable tumors, such as mesothelioma, melanoma, or pancreatic cancer. The RNase inhibitor (RI) present inside almost all cells is the most efficacious sentry to counteract the ribonucleolytic action against intracellular RNAs because it forms a tight, irreversible and enzymatically inactive complex with many monomeric RNases. Therefore, dimerization or multimerization could represent a useful strategy for RNases to exert a remarkable cytotoxic activity by evading the interaction with RI by steric hindrance. Indeed, the majority of the mentioned RNases can hetero-dimerize with antibody derivatives, or even homo-dimerize or multimerize, spontaneously or artificially. This can occur through weak interactions or upon introducing covalent bonds. Immuno-RNases, in particular, are fusion proteins representing promising drugs by combining high target specificity with easy delivery in tumors. The results concerning the biological features of many RNases reported in the literature are described and discussed in this review. Furthermore, the activities displayed by some RNases forming oligomeric complexes, the mechanisms driving toward these supramolecular structures, and the biological rebounds connected are analyzed. These aspects are offered with the perspective to suggest possible efficacious therapeutic applications for RNases oligomeric derivatives that could contemporarily lack, or strongly reduce, immunogenicity and other undesired side-effects.

## Introduction

### RNases

Ribonucleases (RNases) form a very large group of bacterial or eukaryotic enzymes that have been deeply studied in the last 50–60 years (1). RNases catalyze the hydrolysis of a variety of different RNA substrates (2), so that a logical and suitable classification in families may not be easy or immediate. Furthermore, a cell contains a large number of distinct RNases, approaching as many as twenty members, often characterized by different or sometimes overlapping substrate specificities (3). However, notwithstanding this complexity, a useful classification may descend from differentiating intracellular RNases from the ones secreted in extracellular fluids that are also called secretory RNases (1, 4, 5), on which we will focus our attention.

### Pancreatic-Type RNases

The group of secretory RNases has included, since the 60s, an increasing number of members that have been discovered, characterized and classified as the “pancreatic-type” RNases (5, 6). This definition originates from the bovine pancreatic RNase A, a 13.7 kDa and 124 AA residues enzyme that has been the most studied RNase in the past (7–10). The already 20 years old review of Raines still remains an exhaustive milestone that describes many aspects of RNase A, especially its catalytic activity (11). More recently, RNases of the pancreatic-type superfamily proto-type do not refer sometimes to the bovine variant but to human pancreatic RNase (12), called HP-RNase or RNase 1 (6). This variant is not expressed only in the pancreas, but in almost all tissues (13), and displays a highly similar identity sequence as RNase A, although the former is more basic than the latter (14). Both RNase A and RNase 1 can be variably glycosylated at the expense of some Asn residues, especially Asn34, with RNase 1 being more prone to suffer this modification than the former (15, 16). Besides these two members, other important RNases have been included in the mentioned superfamily although they are not secreted by the pancreas: deserving notice are the cytotoxic bovine seminal RNase (BS-RNase) (17), the unique natively dimeric member of the superfamily (18, 19), and also RNase 5 or angiogenin (ANG) (20). ANG is 123 AA residues long and is so called because of its active role in the new vessels formation, but it exerts ribonucleolytic activity against tRNAs as well (21–23). Again, other RNases belonging to non-mammalian species, such as birds or amphibians, are also known (24, 25). Among these RNases, the most studied are the 114 AA residues amphinase and, above all, the smallest variant called ranpirnase or P-30 protein, which is formed only by 104 AA residues (26, 27). This latter variant is commonly called onconase (ONC) because of its remarkable antitumor activity exerted against many cancer types (27, 28). Although not being mammalian, both amphinase and ONC have been included in the pancreatic-type superfamily because of their structural homology with pancreatic RNases, being characterized only by about 30% sequence identity (28). Hence, the main general requisites necessary to belong to the pancreatic-type superfamily are (i) the conservation of a catalytic triad formed by one Lys and two His residues, as H12/K41/H119 is for RNase A, (ii) an elevated homology folding resembling the V-like, or kidney-like shape of RNase A and (iii) the distribution of the basic charged residues that should be almost totally located in the proximity of the active site region (5) to allow an efficacious accommodation of the negatively charged RNA polymer in the RNase active site cavity (29). Therefore, many of the mentioned basic residues are considered “subsites” crucial for an optimal catalysis (11, 30) or they are sometimes considered even as secondary active sites, as was recently reported for human RNase 6 (31). This aspect acquires increasing importance if we recall that RNases can often interact with a polymeric substrate and not only with short oligonucleotides (29). We also point out Arnold's quite interesting review in which the principal features of mammalian and amphibian RNases have been exhaustively compared (25).

### Mammalian and Amphibian Secretory RNases and Their Biological Activities

Up to eight secretory RNase variants, numbered from 1 to 8, have been characterized today in humans. The already mentioned pancreatic RNase 1 is expressed in all tissues, but it is particularly abundant in endothelial cells (32). Its biological activity features are devoted to controlling the extracellular RNA (exRNA) content in the biological fluids, as will be reported below.

RNases 2 and 3 are instead highly basic eosinophil secretory variants that reciprocally share 67% identity but only 32 and 26% with RNase 1, respectively. They are both out of the “pancreatic-type” superfamily because their basic residues are randomly located (5). However, the 134 AA residues long RNase 2 displays an activity against yeast RNA comparable with the one of the pancreatic enzyme (33). RNase 2 is the eosinophil-derived neurotoxin (EDN) that is additionally present in many organs and fluids (34, 35). EDN/RNase 2 elicits Purkinje cells death when released into the cerebellum, but it exerts crucial physiological actions taking part in the innate host defense (36). It is released from eosinophilic granules in response to inflammatory mediators, supporting an immune-modulatory role (37). In particular, cytokines, such as CCL11 and CCL24 can induce RNase 2 and also RNase 3 secretion via the PI3K/MAPK pathway (38, 39). In addition, RNase 2 displays chemoattractant effects by eliciting both dendritic cells' maturation and activation and also exerts activity against both Respiratory Syncytial and Human Immunodeficiency Viruses (36, 39). Indeed, virus infection can trigger eosinophilic RNases release through the toll-like receptors signaling pathways (39). Differently from EDN, the 133 AA residues RNase 3 is eosinophil-specific and is also named the eosinophil cationic protein (ECP) (40, 41). ECP is peculiar in the RNase world: in fact, although it is less neurotoxic than EDN, it exerts remarkable bactericidal effects, promoting the agglutination of bacteria cells and cytotoxicity (42) also when its catalytic activity is nil (43). Several inflammatory stimuli trigger ECP release, so that its serum concentration is used as a biomarker of various Th2-phenotype-associated inflammatory diseases, including asthma, allergic rhinitis, dermatitis and bowel disease (44, 45). Furthermore, and differently from the other wt-RNases, ECP can form amyloid-like fibrils at acidic pH (46).

RNase 4 (119 AA residues) is today the least studied human RNase variant, although its ubiquitous expression suggests for it an important physiological role (36). It is highly selective for uridine RNA sites of cleavage (47, 48), and, similarly to RNase 5, RNase 4 displays angiogenic activity (47, 49). Consequently, it is protective against neuron degeneration by promoting angiogenesis, neurogenesis and neuronal survival under stress (50). Importantly, polymorphisms, and recently, pathogenic mutations have associated RNase 4 with Amyotrophic Lateral Sclerosis (ALS) development (51, 52).

The already mentioned 123 AA RNase 5, or ANG, is considered the oldest pancreatic type RNase member (53). It displays three instead of four paired cysteines with respect to the other RNases (24, 53), and its ribonucleolytic activity, although being very low, is mandatory for its angiogenetic action (36, 54). Its localization is also very important for its somehow contradictory biological functions exerted in different cell compartments and conditions. In fact, in the extracellular space, ANG can trigger growth signaling pathways, such as ERK1/2 and AKT activation, upon its binding to a receptor; however, it is not well-characterized yet (54, 55). Inside the cells, instead, its high affinity binding with the cellular ribonuclease inhibitor (RI) switches off its catalytic and biological activities (55). However, not all ANG molecules are sequestered by RI: indeed, the phosphorylated ones can escape RI and enter the nucleus, accumulating in the nucleolus where ANG stimulates ribosomal transcription and exerts its angiogenetic activity (56). In cancer cells, the increase of rRNA production and angiogenesis definitely enhances cell growth and tumor progression (56). Therefore, ANG inhibition can on one hand counteract tumor growth, but, at the same time, could also promote neuronal damage. In fact, as for RNase 4, many loss of function ANG mutants are associated with ALS and/or Parkinson's disease (57–59). In the contrast of its nuclear activity, ANG can address itself in the stress granules where it splits tRNA into fragments (tRFs) (60). In this case, ANG can perform different functions through an RNA interference-like mechanism (61). Indeed, ANG is known to trigger the formation of cytotoxic tRFs species upon knockdown of RI (62). Consequently, an induced cell death effect can prevail over ANG angiogenetic action. In conclusion, under different conditions ANG can induce either cell death or survival (61, 63).

The 127 AA residues long RNase 6 (64), characterized by two catalytic centers (31), is ubiquitously distributed, including neutrophils and monocytes. RNase 6 is thought to exert an active role in inflammation because its level increases in the urinary tract after infection (65). The antimicrobial properties of this variant are carried out by inducing bacteria agglutination (66). Furthermore, RNase 6, as well as RNase 3, is highly effective against *Mycobacterium aurum* by inducing an autophagy process in the infected macrophages (67).

Finally, RNase 7 and 8 are formed by 128 and 127 AA residues, respectively, displaying high structural similarity, although the former is expressed in the skin but also in other epithelial tissues and organs and can be induced by growth factors, cytokines and bacterial products (68). Conversely, RNase 8 is principally expressed in the placenta but also in the spleen, lung and testis (69), implying the presence of a defense system against pathogens that cross the placenta to target the fetus (70). Importantly, we underline that the most important features of the eight human variants are well-described in the two reviews provided by Sorrentino and, more recently, by the group lead by Boix (39, 71).

From what has been reported, the peculiar and remarkable biological activities exerted by many RNases would not seem at first to be directly related to their ability to hydrolyze RNA. Instead, for the already mentioned BS-RNase, ANG, ONC, and amphinase, at least a minimal ribonucleolytic activity is mandatory to express their biological actions (72), among which the cytotoxicity against malignant cells emerges (49, 73, 74), while since the 70s, BS-RNase has been discovered to be also immunosuppressive, embryotoxic, and aspermatogenic (73, 75–77). Interestingly, the history of the findings related to the antitumor action of many RNases has been well-described by Matousek in 2001 (78).

### Bacterial RNases

Considering their structural and functional properties, we report about four bacterial RNases belonging to the RNase N1/T1 microbial superfamily (79). They are as follows: barnase from *Bacillus amyloliquefaciens* (80–82), binase from *B. pimulus* (82, 83), balifase from *B. licheniformis* (84), and balnase from *B. altitudinis* (85). Barnase is found to be bound with its inhibitor Barstar (80, 81, 86), but when it dimerizes and contemporarily forms a dibarnase immuno-derivative it exerts a remarkable antitumor activity against many cancer cell types (87–89). Binase is natively dimeric (83, 90), and possesses remarkable cytotoxic and antiviral activities against transformed myeloid cells and fibroblasts, also against SiHa cervix human papilloma virus-infected carcinoma cells, without inducing immune response (83, 91–93). In addition, a molecular mechanism that is carried out without catalytic degradation of RNAs has been suggested by Ilinskaya et al. to explain some binase anti-tumor effects. Indeed, binase is reported to interact with KRAS, stabilizing the inactive GDP-bound conformation of RAS, thereby inhibiting MAPK/ERK signaling (94). Balifase is then the most stable variant of this group and is not natively dimeric, but it combines parts of binase and barnase features (84). Balnase is almost identical to binase except for its A106T mutated residue (85). However, its biological activities, as well as the ones of balifase, have not been investigated enough yet.

RNases belonging to the T2 family, whose human variant is named RNASET2, also deserve to be mentioned for their remarkable biological activities: they are found in bacteria, plants and viruses but also in animals, and they exert their enzymatic activity at pH values around 4–5—indeed lower than neutral pH, around which the majority of RNases are active (95). RNASET2 is secreted by damaged tissues, exhibits chemotactic activity and initiates immune response(s): in fact, recombinant RNASET2 injection induces fibroplasias, connective tissue remodeling and the recruitment of infiltrating cells expressing macrophage markers (96). Furthermore, humans lacking or carrying RNASET2 mutations suffer neurological disorders or even misfunction in the immune system (97).

### Secretory RNases as Natural Scavengers of Extracellular RNA (exRNA)

The ca. 20 active RNases present in almost all mammalian cell types process RNA into mature forms to regulate RNA turnover and metabolism and properly tune the associated cellular processes (98, 99). Additionally, RNases can also work as alarming sentinels to block several cellular dysfunctions: indeed, they can act as immune regulators or agents inducing tissue repair and remodeling, epithelial barrier protection, body fluid sterility and exRNA clearance (39). Regarding this latter aspect, exRNA promotes the activation of proteases that trigger the blood coagulation factors XI and XII, while a RNase A pretreatment can delay occlusive thrombi formation (100). Moreover, exRNA mediates the endothelial brain permeability and RNase A treatment reduces vessel occlusion, preventing brain edema (101). Furthermore, the *in vivo* administration of RNase 1 reduced pathological parameters that are characteristic for ischemia/reperfusion injury, thus improving functional myocardial recovery (102). Finally, considering that exRNA is able to increase inflammation by stimulating leukocyte adhesion, transmigration and mobilization of pro-inflammatory cytokines (103), secretory RNases can become crucial modulators of physiological cell functions by acting as natural exRNA scavengers.

Moreover, it has been recently discovered that exRNAs, especially non-coding ones secreted from tumor cells, can act as signals that modify the cell microenvironment and favor tumor progression and metastasis formation (104). The largest part of exRNA released in body fluids is encapsulated inside extracellular vesicles and exosomes or is associated with high density lipoproteins (105, 106). Thus, even if in these vesicles exRNA is more protected, most secretory RNases are able to cross the membrane, as it is reported hereinafter, thus exerting an anti-tumor activity to counteract metastatic processes also at the extracellular level (107).

In general, human secretory RNases also display anti-inflammatory, antibacterial and antiviral actions, or induce immune response when a large amount of ds-RNA is generated by pathogens (53, 108). Additionally, RNase 1 is active against DNA:RNA hybrids, and ds-RNA rather than ss-RNA (12, 109, 110), and, to this end, the conservation of its Gly38 residue is crucial to maintaining the full catalytic activity against duplex RNAs (111). Overall, many steps forward have been performed in the comprehension of the antimicrobial effects ascribable to many RNases, and promising results suggest their potential use despite the classic antibiotics that lose efficacy upon the development of resistance (31, 44, 112).

In summary, almost all the biological activities displayed by the mentioned RNases are strictly dependent on their ribonucleolytic activity vs. ss- and ds-RNA, necessary to regulating the extracellular RNA level (36, 39, 100, 113).

The secreted RNases endowed with relevant biological activities do not display their action only extracellularly, but they are often able to enter the cell to exert their peculiar activities against intracellular RNAs. However, at this step, many difficulties indeed emerge, such as the necessity to have a favorable interaction with cell membranes, a subsequent release in the cytosol and the possibility to evade RI (114). These issues are more extensively described in the next chapters, together with the ability of RNases to form supramolecular structures. The formation of oligomeric moieties can become in fact advantageous for many RNases in terms of acquiring new activities or potentiating pre-existing ones, as well as minimizing undesired interactions, or also reducing adverse side effects *in vivo* (115–117).

## Crucial Steps for Cytotoxic Extracellular RNASES to Exert Their Activity

### Interaction With (Malignant) Cell Membrane and Cell Entering Upon Endocytosis

The main obstacle for extracellular RNases to exert their antitumor action is represented by the possible difficulty to be cellularly internalized. This occurs through endocytosis (118) and only if a fruitful interaction with the cell membrane occurs. However, the possibility for ONC to be internalized with the mediation of a receptor has been proposed and debated as well (119, 120). Sundlass et al. compared the ability of RNase 1, RNase A and ONC to interact with lipid bilayers, revealing that either electrostatic forces or specific interactions determining the time spent by an RNase near the cell surface are critical for its internalization (121). In addition, Notomista et al. demonstrated that cytotoxic native or engineered dimeric RNases strongly affect membrane aggregation, fluidity and fusion (122). Considering that the most desired biological activity of RNases is to be selectively cytotoxic against malignant cells and that these cells possess a more negatively charged membrane than normal ones, the basicity of each RNase is likely the most important feature necessary to win this challenge. However, the basic net charge *per se* is not enough for a RNase to be internalized in the cell. In fact, the specific orientation of a RNase molecule can become crucial for a successful approach to the cell membrane: therefore, the selection of the most favorable RNase-membrane interaction patch has been the object of many studies focused especially on ONC and BS-RNase, but also on RNase 1 and RNase A (12, 121–123). In particular, the natively dimeric BS-RNase assumes the most advantageous orientation for its internalization when it faces both N-termini toward the cell membrane (122), and the structure of BS-RNase shows that both N-termini actually adopt the same orientation (19). Furthermore, the G38K-BS-RNase mutant, being endowed with an additional cationic key-residue oriented in the same direction of the N-terminus, interacts with the membrane more strongly and is even more cytotoxic than the wild type (124, 125). Hence, the the orientation of the basic charges could also affect the cytotoxic potential of other RNases and of their oligomers. Other studies showed instead that BS-RNase approaches the membrane differently from ONC (121) but similarly to RNase A and RNase 1 (121, 122). RNase 1 in turn showed itself to be the monomeric variant accompanied with the highest propensity to be internalized in cells (12). Moreover, RNase cellular internalization can be considered also residue-specific: in fact, wt-ONC is less efficiently internalized than its so called “R-mutant,” in which all lysine residues but the two crucial for catalysis are replaced by arginine (126). It has to be underlined, however, that if the RNase basic net charge is randomly increased, the advantage represented by a favored internalization may be counteracted once in the cells by the RNase affinity increase toward the negatively charged RI (127).

Finally, and interestingly, we note that BS-RNase has a high binding affinity also toward the extracellular matrix (ECM), since its cytotoxicity against CHO cells grown in suspension is quite lower than the one exerted vs. the same adherent cell subtype (128).

### Intracellular Routing of RNases

Once they have entered the cell, RNases must overpass other obstacles to exert their activity. Some of these issues have been well-presented in another review contributed by Arnold (129). The cytotoxic potency of different RNases is certainly due to optimal cell membrane interaction and endocytic internalization, but also to their resistance against proteolytic degradation in the endosomes/lysosomes/trans Golgi and their fruitful release into the cytosol (118).

Some discordant data are present in literature regarding the actual internalization mechanism of RNases: in fact, while Haigis and Raines demonstrated that ONC, RNase A and its G88R variant are internalized in early endosomes of HeLa and K562 cells by a clathrin- and caveolae-independent mechanism (120), Rodriguez et al. indicated that Jurkat cells endocytose ONC through a dynamin-dependent route, presumably following a clathrin/AP-2-mediated endocytic pathway (130). However, these controversial data suggest that RNases go through diverse routes to cross the membrane of different cell lines. In the RNases routing toward their final cytosolic destination, the first step is their delivery to early endosomes, while instead the features of the subsequent intracellular routing steps have not been completely understood so far. BS-RNase is internalized in endosomal vesicles either in normal or malignant cells; but only in the latter ones, where it is cytotoxic, does it reach the Golgi apparatus, i.e., the intracellular station, before its cytosolic delivery (131, 132). In addition, a BS-RNase variant characterized by a C-terminus engineered with a key-sequence that is useful for its localization in the endoplasmic reticulum is not cytotoxic because it is unable to be released in the cytosol to elicit its anti-tumor activity (131). Furthermore, when two RNase 1 mutants designed to evade RI were fused with a scFv fragment for human CD7 antigen to be delivered into leukemic cells, they were able to bind to T cell surface and to be internalized, but they were not cytotoxic. This is because they were delivered in the lysosomal compartment and there degraded. Instead, cytotoxicity was restored when they were internalized by transfection (133). In contrast, the human immuno-ErbB2-RNase 1 fusion protein was internalized in SKBR3 cells, and its direct transfer from endosomes to the cytosol was demonstrated. In this case, cell death occurred through apoptosis with an IC_50%_ of 12 nM (134). More recently, an interesting strategy to intracellularly internalize RNases was proposed: three cationic amine-reactive linkers were attached to RNase A, and the stability of these conjugates was pH-dependent. Therefore, the endocytic vesicles' acidic environment led to the release of high cytosolic amounts of RNase A to make its concentration high enough to overcome the RI binding capacity and become cytotoxic (135).

However, RNases can have distinct but not always successful outcomes in several cellular organelles, such as endosomes, lysosomes, ER and the trans-Golgi network (136). Thus, many studies have been performed to unveil the intracellular routing of extracellular RNases once internalized by endocytosis, and the relative results indicated that diverse RNases, such as ONC or BS-RNase as well as RNase A, RNase 1, or ANG follow different cellular pathways within one another, as described or proposed in several reports (32, 118, 120, 128, 130–132, 135).

Finally, it has to be remembered that the capability to exert their activity in the nucleus is crucial for some RNases: indeed, ANG/RNase 5 needs to be enzymatically active (72) but also to enter the nucleus to exert its fundamental angiogenic activity in both normal and cancer cells (56, 137). To do so, ANG exploits its surface loop involving the R31-R32-R33 Arg-triplet, whose replacement provokes the block of either its nuclear import or angiogenesis (138). Notably, insertion(s) of nuclear localization sequence(s) (NLS) involving Arg residues located in loop(s) present in RNase A or RNase 1 variants make them able to enter the nucleus and exert angiogenetic (139) or cytotoxic activities (140, 141), respectively. This evidence suggests that different RNases located in the same compartment can address their action against different RNA targets to trigger diverse biological reactions. Then, considering the human nature of RNase 1, its nuclear activity becomes advantageous in terms of obtaining cytotoxic variants devoid of any immunogenicity.

### Evasion From the Cytosolic RNase Inhibitor

As already mentioned, a huge obstacle for an RNase to be actually active in the cell is represented by the interaction with RI. This 50 kDa protein was firstly extracted from the rat liver while the human variant was isolated from placenta (142), and its interaction with RNases was firstly detected with ANG (143). RI is a negatively charged horseshoe-shaped and leucine-rich macromolecule that is ubiquitously expressed in almost all cells (114). RI was considered for many years to be present only in the cell cytosol, but more recently its presence has been detected also in mitochondria and nuclei (144). All RI biological functions have not been completely clarified, but, considering its numerous cysteine residues, its contribution to the redox cellular homeostasis has been stated (145). RI can form very tight complexes with RNase A (146) and with RNase 1 (147), ANG (148) and EDN (149). RI interacts also with RNase 7 (150) and is highly conserved in the cells of many mammalian species, but is present also in non-mammalian ones (151). Most of the RNase-RI complexes characterized so far are not dissociable because their K_d_ values fall into the pico- to femtomolar range (152). The RNase-RI complex structure(s) explain(s) why RNase activity is inhibited, being the enzyme moiety caged inside the RI cavity (153).

ANG can sterically evade the cytosolic interaction with RI when it undergoes phosphorylation at some Ser/Thr residues that are crucial for RI binding (55). In this way, it can be internalized in the nucleus to exert its angiogenetic activity. Instead, ONC exceptionally evades or extensively lowers its affinity for RI because it is devoid of the K7 and G88 key-residues allowing, in RNase A, the interactions necessary to form the tight RNase-RI complex. For this reason, ONC can actually display its remarkable cytotoxicity (154). Hence, site-specific mutagenesis approaches made also RNases of mammalian origin capable of evading RI and exerting their cytotoxic potential (155–157). This could be considered not completely true if we remember that many years ago, Ledoux found that wt-RNase A can be active against tumors either *in vitro* or *in vivo* (158–160). However, we must underline that high RNase A doses, not <200 mg/mouse (159), or concentrations not lower than 2 mg/mL (~145 μM) in the cell culture medium (160), were used in those cases. Therefore, even if all RNase A molecules did not enter the cells, these doses certainly overpassed the cytosolic RI concentration, that is about 4 μM (161). Hence, the exceeding RNase A amount should be free to exploit RI saturation and exert cytotoxic activity, in line with that reported by Liu et al. (135). We recall that, beyond ONC, mammalian BS-RNase is also cytotoxic: indeed, being natively dimeric, it can sterically evade RI (162). It is well-known, in fact, that the monomeric BS-RNase derivative, although retaining its ribonucleolytic activity, definitely loses its cytotoxicity because it is sequestered by RI (163, 164). Finally, another way to make potentially active RNases actually cytotoxic could be to silence the RI action (165), even though the sensitivity to RI is not the unique factor determining cell cytotoxicity. To this point, conflicting results support opposite arguments: certainly, it has been demonstrated that non-cytotoxic RNases are unable to affect HeLa cells viability also after being deprived of RI (166). On the contrary, non-covalent artificial dimers of RNase A, although being RI-sensitive (167), were found to be cytotoxic against some malignant leukemia cell lines at a concentration of about 20 μg/mL (168), even if they were definitely inactive against pancreatic cancer cells (125).

From what has been reported, we can envisage that if an RNase is induced to oligomerize, and, thus, to be bulkier than its native monomer, this would augment the charge density of the enzyme moiety to help its internalization in tumor cells. Furthermore, the increased steric hindrance would help to evade the RI interaction, as BS-RNase does. This strategy, and what it is correlated with, will be presented and discussed in the next chapters.

## RNase Oligomerization: A Strategy to Obtain Stable and More Active RNase Derivatives

Each step necessary to exert their biological action could represent a huge obstacle for RNases. Hence, point mutations as well as post-transduction or also *in vitro* modifications might be helpful to overcome these barriers. Moreover, in this context, a controlled induction of protein self-association leading to natural or artificial RNase oligomers may represent a fruitful strategy to be promoted or, conversely, underwent by the organism to obtain RNase derivatives that exert remarkable biological activities. In line with this argument, RNase or, generally, protein oligomerization, can occur spontaneously or can be induced also by the cell environment or by an *in vivo* context (117, 169, 170). Again, oligomerization can be provoked artificially *in vitro* to obtain a controlled plethora of products that can be characterized in light of the desired goal.

### Covalent Oligomerization of RNases

Artificial oligomerization can be induced to form covalently linked derivatives upon the reaction of a protein with bifunctional or multifunctional cross-linkers. In this way, stable hetero- or homo-oligomers can be produced, but with the concomitant modification of one or more AA residue(s). Dimerization or oligomerization can be obtained also with conditions favoring a spontaneous protein self-association. Again, the cross-linking of the subunits of a protein can occur spontaneously or in response to a cell signal, in membrane proteins or cell factors upon undergoing photochemical events, phosphorylation, apo/holo transitions, or even other post-transduction modifications. To date, within mammalian RNases, only BS-RNase is known to be a native homo-dimer thanks to the formation of two antiparallel disulfides involving the Cys31 residue of one subunit with the Cys32 of the other, and vice-versa (18). These two residues are instead different from cysteine in all other known RNases. Therefore, many strategies have been followed to induce a RNase to dimerize or oligomerize by forcing it to react with the appropriate cross-linking reagent(s). It should be taken into account, however, that any chemical modification can somehow modify the properties of native RNase and, thus, might negatively affect its biological activities. Hence, a balance between desired modifications and an excessive unwanted affection of the native properties is mandatory to obtain derivatives accompanied with fruitful enzymatic and biological activities.

#### Covalently Linked Homo-Oligomeric RNases and Their Biological Activity

**Figure 1:**
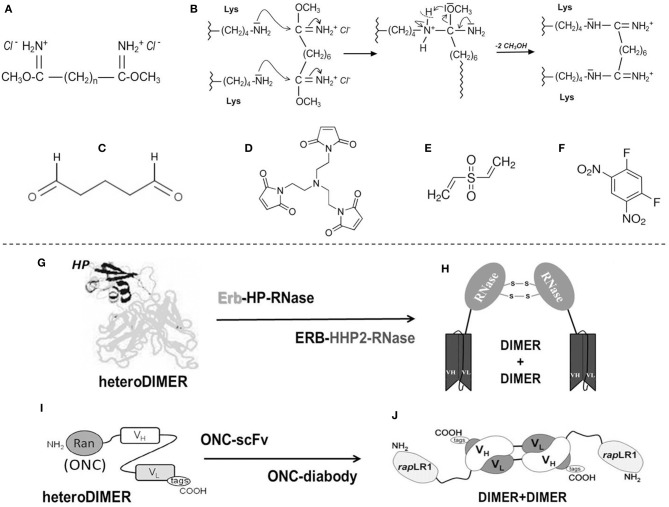
Chemical cross-linkers mostly used with RNases and main immuno-RNases oligomeric derivatives. **(A)** Diimidoesters (171); **(B)** Mechanism of reaction of RNases with diimidoesters; **(C)** glutaraldehyde; **(D)** trifunctional maleimide (172); **(E)** divinylsulfone (DVS) (173); **(F)** difluorodinitrobenzene (DFDNB) (174); **(G)** Immuno-HP-RNase-heterodimer: the HP moiety (black) and the Erb2 one (gray) (175); **(H)** HP-RNase diabody (176); **(I)** Immuno-ONC-heterodimer (177); **(J)** ONC (rap)-diabody (177).

RNases can be artificially associated by exploiting cross-linking reactions with various bifunctional or multifunctional reagents. The most used cross-linkers are mentioned below. This involves especially RNase A and RNase 1, and will be accompanied with the description of the positive or negative rebounds on the relative catalytic and/or the antitumor activities of the products obtained:

- Diimidoesters or dialdehydes (Figures 1A–C), i.e., bifunctional cross-linkers, can react with the Lys residues of a protein. These reagents display two terminal reactive groups separated by a variable number of unreactive methylene (–(CH_2_)_n_-) spacers. As concerns RNase A, its reactions with dimethyl-adipimidate, dimethyl-pimelimidate or, especially, dimethyl-suberimidate, i.e., reagents displaying spacers with different “n” values (Figure 1A), allow differently sized stable dimers or oligomers (171, 178) to be formed, often accompanied with higher enzymatic activity than the native monomer. Cross-linkers are advantageous to produce protein homo-oligomers or, alternatively, a cationized and thus more cytotoxic complex, by adding bulky amines or polyamines through the covalent mediation of the diimidoester (179– 181). The cross-linking can be reversible if the spacer contains a disulfide bond that becomes scissile once the cytosolic reducing environment is reached (182). The reactivity of the Lys nucleophilic residues for the two imides of the cross-linker (Figure 1B) can be more efficiently controlled than with dialdehydes like glutaraldehyde (Figure 1C), and this allows the processed protein to maintain its overall charge unmodified (171, 180). An important undesired limitation is represented by the susceptibility to the cross-linker of the active site Lys residue unless it is not protected by phosphate, thus driving toward a percentage of inactive products (171, 183). However, upon properly tuning the reaction conditions, amounts of RNase A dimers characterized by enzymatic and cytotoxic activities definitely higher than native RNase A can be produced (184, 185). Again, beyond 20% yield of active RNase A dimers, dimethyl-suberimidate (Figure 1B) allowed the formation of amounts of covalently linked trimers and traces of higher-order oligomers, all characterized by a relevant cytotoxic activity against cells of the uterus cervix squamous carcinoma (183). Beyond polyamines, RNases can be complexed also with differently sized PEG moieties: indeed, a RNase 1 PEG-derivative is known to inhibit tumor growth in mice (186), while PEG-conjugated RNase A oligomers are cytotoxic against transplanted melanoma in mice as well (187).
- To obtain active RNase oligomers, the bifunctional N-substituted maleimide derivatives displaying spacers of variable length are certainly useful (182). These reagents induce the cross-linking between a free Cys sulfhydryl and the maleimide to form a reversible adduct if the spacer contains a scissile disulfide bond. The presence of a protein free –SH group is mandatory to make this cross-linking productive. Unfortunately, RNases do not display free cysteines, and, consequently, the reaction can occur only upon introducing a free Cys residue upon mutagenesis (172). Maleimides can also be trifunctional (Figure 1D), or even more, in this way producing covalently linked trimers and/or larger oligomers. Indeed, the group led by Raines succeeded in obtaining many trimers by coupling cysteine-free RNase A, RNase 1, BS-RNase, or ONC mutants with tris(*N*-maleimidoethyl)amine (172). For all these RNases except for ONC, many mutants displaying a Cys in spite of the Gly88 residue were produced. The mutation of G88 of the wild type is required because this residue allows the proper accommodation of a RNase molecule into the RI cavity to form the already mentioned tight RNase-RI complex (152, 153). Instead, since ONC lacks Gly88, the S72C-ONC mutant was produced to allow the formation of the trimer upon cross-linking the protein with the trifunctional maleimide. All RNase trimeric adducts but the ONC one increased their antitumor activity, either *in vitro* or in mice, with respect to their inactive or less active wt-monomers. Only the trimeric S72C-ONC showed instead to be less cytotoxic than its definitely active wt-ONC monomer (172). However, the advantage to obtain a bulky ONC trimeric derivative resides on its very probable steric inability to be filtered by the kidneys' glomeruli and, as a consequence, on its increased circulating half-life. Indeed, this may represent a crucial advantage in the perspective to use these products in cancer therapy, considering that the monomeric wt-ONC displayed renal toxicity *in vivo*, although this undesired effect was reversible upon discontinuing the treatment (188).
- Zero-length dimerization: RNases dimers or oligomers can be formed also upon interaction with carbodiimides (R_1_-N = C = C-${\text{R}}_{2}^{+}$), i.e., dehydrating molecules, such as *N*-(3-Dimethylaminoisopropyl)-*N*′-ethylcarbodiimide (EDC), that can induce the formation of a “zero-length” dimer(s) or larger oligomer(s) characterized by isopeptide bond(s) involving the side chain of the amino group of Lys and the carboxylate group of Asp or Glu residues (189). These reagents can covalently fix previously formed oligomeric aggregates and contemporarily avoid unwanted charge modifications or insertions of chemicals in the protein complex. Supramolecular RNase zero-length adducts can be produced also by heating the protein up to 85°C under *vacuum* (190). This method allowed RNase A to form novel isopeptide bonds between Lys and Glu or Asp residues upon inducing heat-*vacuum* dehydration. This condensation reaction affects principally, but not exclusively, the RNase A E9 and K66 residues (190, 191). However, these derivatives showed a definitively reduced enzymatic activity and inactivity against cancer cells unless they were strongly cationized with polyspermine. This is probably due to the excessive number, at least three, of the affected Lys residues and, thus, by the scarce specificity of the cross-linking reaction that drives toward a mixture of heterogeneous products (191). However, it must be mentioned that the strong cationization allowed the other RNase adducts to gain cytotoxicity but also aspermatogenic effects (181, 192–194).
- More recently, oligomers have been produced also upon mixing at 37°C RNase A with metallic derivatives commonly used as chemotherapeutic agents, such as cisplatin, or also carbo- or oxaliplatin (195, 196). The yield of RNase A-cisplatin oligomers remarkably increases with the cisplatin/protein ratio (195), while with the other Pt adduct yields are definitely lower (196). Unfortunately, these adducts are endowed with scarce ribonucleolytic activity and lack cytotoxicity principally because the catalytic H119 residue is directly involved in the linking with the metallic moiety (195, 196). Hence, this result helps to explain why cisplatin and/or similar adducts display a reduced efficacy and undesired side-effects in therapy: in fact, this is because they very probably affect proteins, such as here RNase A, or *in vivo* serum albumin as well, at a higher extent than the desired DNA target.
- RNases can be cross-linked also by using the bi-functional cross-linkers divinyl-sulfone (DVS, Figure 1E) or difluorodinitrobenzene (DFDNB, Figure 1F), which are specific for His or Lys residues, respectively (173, 174). These cross-linkers lack spacers and can cross-link only residues residing very close to each other, or they can covalently stabilize preformed oligomers. Indeed, they are extremely useful for characterizing the mechanism of formation of non-covalent supramolecular structures produced by BS-RNase, RNase A or ONC (173, 197–200), but they can be used only for analytical purposes because they totally inactivate the resulting products by involving active site residues.

#### Covalently Linked or Fusion RNases Hetero-Oligomers and Their Biological Activities

- Asymmetric bifunctional reagents are certainly useful in overcoming the lack of protein-free Cys residues: in fact, they can covalently link antibodies, or parts of them (light/heavy chains), with proteins, protein domains, toxins or whatever displays biological interest (Figures 1G,I). They can be, on one side, maleimides or succinimides sometimes displaying dithio-derivatives in the spacers, combined in the other terminus with imidoesters, diones, thiones, or 2-iminothiolane. One reagent often used to achieve a covalent hetero-dimerization is 1-(3-((2,5-Dioxopyrrolidinyl)oxy-carbonyl)phenyl)-1H-pyrrole-2,5-dione (MBS), but many others are available (201). Some of them have been tested with proteins different from RNases (202), but asymmetric reagents have been used to form active hetero-adducts involving RNases as well: in fact, ONC displayed a remarkable antitumor activity against breast cancer or brain glioma both *in vitro* or in mice upon its chemical conjugation with Trop-2 (or EGP-1), or, alternatively, with a chlorotoxin (203, 204). Again, strong specific anti-tumor effects exerted by both LL2 or anti-CD22 monoclonal antibodies covalently linked to ONC significantly increased the life span of animals displaying non-Hodgkin B-cell lymphoma (205). Some other chemically cross-linked EGF-RNase or Immuno-RNase tumoricidal adducts have been produced since the 90s with RNase A (206, 207) or RNase 1 (208). The list of all the mentioned heterocomplexes are reported in Table 1 together with the ones obtained as immuno-fusion products that are discussed hereafter.
- Alternatively, protein engineering permits cloning and expressing fusion homo- or hetero-fusion dimers as well as fusion immuno-protein conjugates (Figures 1G–J). The cDNA encoding the desired RNase is fused with genes of heavy or light single-chain antibodies. The same strategy can be followed with diabodies (Figures 1H,J), which are non-covalent dimers of single chain antibody fragments (scFv) consisting of variable regions of heavy and light chains (V_H_ and V_L_) connected by a small peptide linker (175, 201). If desired, additional peptide spacers of various lengths and nature can be inserted between the proteins (210, 218, 222). In this way, a natural covalent link binds the two proteins, thus permitting the expression of the complete adduct without requiring subsequent chemical modifications. Again, in some cases, as it is for diabodies, the hetero-dimeric immune-RNase adduct has been considered as a new entity that can be further dimerized to form a more active tetramer, as it was performed with both RNase 1 and ONC (Figures 1H,J) (176, 177, 216). Additionally, a fusion protein composed of two ONC molecules, each fused to the N-terminus of the V_L_ of an anti-CD74 humanized antibody, was demonstrated to exert an excellent therapeutic efficacy against CD74^+^ tumors either *in vitro* or *in vivo* (217).

**Table 1:** Chemically linked or recombinantly fusion-produced antitumor-active secreted immuno-RNases.

**Immuno-RNase conjugate**	**Chemical/fusion conjugation**	**Ligand = linker/spacer**	**Diabody Y/N**	**Cell/target and/or human cancer counteracted**	**References**
Transferrin(Tf)-RNase A	Chemical	Peptide	N	Tf-Receptor Leukemia	(108)
Anti-TfR-RNase A	Chemical	Monoclonal Antibody	N	Tf-Receptor Glioma	(108)
scFv CD5-RNase A	Chemical	Succinimidyl-pyridyl-thiopropionate (SPDP)	N	K562 and Jurkat-Leukemia; U251-glioblastoma cell lines	(206)
EGF-RNase A	Chemical	Succinimidyl-pyridyl-thiopropionate (SPDP) and 2-iminothiolane (2-IT)	N	Many squamous carcinoma and breast cancer cell lines	(207)
RNase A-RNase A tandem	Fusion	(SG)_3_S/SGRSGRSG/GP_n_G	N	K-562 leukemia cells	(209)
EGF-RNase 1	Chemical	Succinimidyl-pyridyl-thiopropionate (SPDP) and 2-iminothiolane (2-IT)	N	Many cancer cell lines	(208)
scFv-ErbB2-RNase 1	Fusion	GSPEFM peptide	N	SKBR3 and MDA-MB453 breast cancer cells; A431 epidermal carcinoma cells	(210)
scFv ERB-HHP2-RNase1	Fusion	SS(G_4_S)_2_GGS linker AAASGGPEGGS junction peptides	N	SKBR3 and MDA-MB453 breast cancer cells; A431 epidermal carcinoma cells	(176)
scFv-Erb-hcAb-RNase 1	Fusion	AAASGGPEGGS peptide linker	N	SKBR3 and MDA-MB453 breast cancer cells; A431 epidermal carcinoma cells	(211)
AntiNCL scFv4LB5-RNase1	Fusion	SGGGGSGGGGSGGS linker AAASGGPEGGS spacer	N	MDA-MB-231/436, BT549 breast cancer cells; SW 620 colon adenocarcinoma cells; *In vivo* nude mice MDA-MB-231 triple negative breast cancer	(212)
FGF Nterm-RNase 1	Fusion	LPALPEDGGS peptide linker	N	Many cell lines	(213)
IL2-RNase 1	Fusion	Not specified	N	MJ, OKM, and MOLT-3 leukemia cells lines	(214)
αCD30scFv-RNase1-Fc αCD30scFv-Fc-RNase1	Fusion	AAASSG peptide linker	N	Karpas-299 Lymphoma cell line HEK 293T human embryo kidney cells	(215)
scFv CD7-RNase 1 variants scFv CD7-ONC	Chemical/Fusion	Succinimidyl-pyridyl-thiopropionate (SPDP)/TRHRQPRGWEQL furin-sensitive peptide	N	K-562/Molt 3/SEM K2 myeloid leukemia cell lines	(133)
Chlorotoxin-ONC	Chemical	Succinimidyl-pyridyl-thiopropionate (SPDP)	N	Glioma U251 and SHG-44 cells	(204)
scFv LL2-ONC	Chemical	Succinimidyl-pyridyl-thiopropionate (SPDP)	N	Daudi lymphoma in mice	(205)
anti-CD22 scFv SGIII-ONC	Fusion	GGGGS peptide	Y	CA46 + Raji Burkitt lymphoma cells Daudi lymphoma cells Jurkat leukemia cells; tRNAs	(216)
Anti-EGFR-scFv IZI08-ONC	Fusion	GGGGS or (G_4_S)_3_ peptides	Y	A431/Raji/HNO and FaDu oro-pharyngeal/CAL27 tongue/MCF7 breast cell lines + *In vivo* A431 Nude mice	(177)
2L-ONC-hLL1-γ4P (S228P)	Fusion	(G_4_S)_3_ peptide linker	N	Daudi/Raji and MC/CAR lymphocytes + SCID or BALB/c *in vivo* mice	(217)
4D5MOCB-Album-_O_-ONC	Fusion	Circular<RKRRC_S−S_CAEAE<peptide	N	HT29 Colorectal carcinoma and A375 melanoma cell lines	(218)
Anti-EGFR scFv IZI08-Dengue-ONC	Fusion	MVDRGWGNGCGLFGKGGIV Dengue peptide	N	HNO97/HNO211/HNO410 oral, A431 epidermal, and MCF7 breast carcinoma cells	(219)
ONC-DV3	Fusion	PFV linker	N	MDA-MB-231/MCF7 breast PC-3M-1E8/PC-3M-2B4 prostate PG-BE1/PG-LH7 lung cancer	(220)
Transferrin Nterm(TFn)-ONC	Fusion	(G_4_S)_3_ peptide linker	N	HepG2 hepatocarcinoma HeLa cervix carcinoma cell lines	(221)
ONC-AD2-IgG-ONC	Recombnt + Chemical	Redox Peptide GSGGGGSG + HisTag	Y	Trop2/CD20/CD22 Liver HCC1395/Breast MDA-MB-231	(203)
sFv V_L_/V_H_-ANG	Fusion	EGKSSGSGESKEF or (GGGGS)_3_ peptides	N	Colorectal HT29, MDA-231 breast cancer, ACHN kidney carcinoma cells	(222)
CD64 scFv-H22-ANG mutants	Fusion	G85/86R and/or Q117G mutations H22-fragm, linker not specif	N	Yeast tRNA/HL-60 and L-540y leukemia cells	(223)
scFv MJ 7-ANG/ ANG-MJ 7/ scFv MLT 7-ANG dimer	Fusion	(G_4_S)_3_ peptide linker-MJ 7 direct linking with MLT 7	Y	Daudi/Jurkat/Raji cells and HuT 102 cutaneous lymphoma cells	(224)
EDN-antiTranferrin-scFv	Fusion	AKKLNDAQAPKSD peptide	N	A431 epidermal carcinoma/ K562 leukemia cells	(225)
scFv Diphteria Toxin (DT)-BS RNase	Fusion	KDEL BS-elongation peptide + N-term DT Linker	N	A431 epidermal/KB epithelial carcinoma cells	(226)
scFv glycoprotein A33-RNase T1	Fusion	HisTag/linker not specified	N	SW1222 colon carcinoma gpA33 positive cells HT29 colorectal and MCF-7 breast gpA33 negative cells	(227)
scFv 4D5-dibarnase	Fusion	Peptide + HisTag	N	DNA fragmentation/apoptosis many cell lines	(87)
scFv 4D5-dibarnase	Fusion	Peptide + HisTag	N	Cell apoptosis Breast cancer—mice	(88)
scFv 4D5-dibarnase/colloid gold-barstar complex	Fusion	Peptide + HisTag	N	Endosomes/lysosomes Ovarian and breast carcinoma SKOV-3 and BT-474 cells	(89)

Following these strategies, several Immuno-RNase or Immunotoxin-RNase complexes have been produced and used as anticancer agents (175, 228) also with RNase A. Instead, the group of D'Alessio and De Lorenzo chose to couple human RNase 1 with the human anti-ErbB2 (Figure 1G) (175, 210, 211, 229). The resulting heterodimeric ERB-hRNase adducts showed to be definitely more cytotoxic against breast cancer and induced less resistance and cardiotoxicity than a humanized anti-ErbB2 monoclonal antibody used as a chemotherapeutic agent (175). Furthermore, the tetrameric adduct, i.e., the dimer of the hetero-dimer, called ERB-HHP2-RNase, was displayed to be even more cytotoxic than the simple ERB-hRNase adduct (176). Again, some RNase 1 mutants designed to evade RI, and fused to a scFv fragment specific for CD7, reduced the viability of human T leukemic cells (133). More recently, an anti-nucleolin(NCL)-immunoRNase 1 derivative was displayed to be active against triple-negative breast cancer not responding to treatments available so far (212).

The protein-fusion technique has been applied also to ONC to conjugate and express it with many diverse adducts (Figures 1I,J), such as various antibody fragments, human serum albumin, dengue virus-derived peptide, and more recently with the N-terminal domain of transferrin (177, 216, 218, 219, 221, 230). All these adducts displayed augmented cytotoxicity *in vitro* against many cancer cells, and in some cases also in mice (177, 218). This augmented activity was often accompanied with a lower propensity to undergo renal filtration, as indicated by the results obtained with membranes mimicking the kidney glomerular barrier. Then, another crucial advantage offered by human Immuno-RNases over microbial or plant immunotoxins is the lack of immunogenicity or of non-specific binding and toxicity that usually also affects normal cells. These undesired events drove in some cases even toward fatal events during clinical trials (231). Indeed, De Lorenzo and D'Alessio clarified that the immune-RNase 1 fusion proteins were not only non-toxic outside the tumor cells but also non-immunogenic (228). Again, as reported in Table 1, the protein-fusion strategy against cancer has been used many other times with RNase 1 (213–215) and ONC (220), but also with BS-RNase (226) and EDN (225), producing cytotoxic derivatives. Also Immuno-ANG derivatives have been produced, and the effects on their cytotoxic activity induced by proper ANG mutations (223) or by different linkers introduced in the supramolecular adducts have been compared (222). Again, ANG was driven also toward a diabody-conjugated dimer that was revealed to be definitely more cytotoxic than the monomer (224). Notably, bacterial barnase and fungal RNase T1 have also been derivatized as immunotoxins to become capable of being internalized in cancer cells and exert a remarkable cytotoxic activity (87–89, 227). Finally, in the last decade, RNase A has also been dimerized by cloning and expressing it as a tandem derivative in this way:

Monomer1-C-term—peptide linker—N-term-Monomer2

Contrarily to the inactive monomer, this fusion-stabilized RNase A derivative became definitely cytotoxic against K562 human leukemia cells (209), although it was revealed to be 1:1 complexed with RI (232). This apparently contradictory result could be explained by considering that each RNase A fusion moiety contains one active site, and one of them could be oriented in a way to actually exert its catalytic and cytotoxic actions. To this regard, we underline that the structure of the fusion RNase A—RNase A derivative (see Figure 3B) is not in contrast with this explanation (209, 232). All the fusion-produced immuno-derivatives here mentioned are listed in Table 1. Again, many reviews have been focused on the selective cytotoxic action of Immuno-RNases against tumor cells (108, 175, 238), as it is for the recent and updated one of Jordaan et al. (239).

### Non-covalent RNase Oligomers

**Figure 2:**
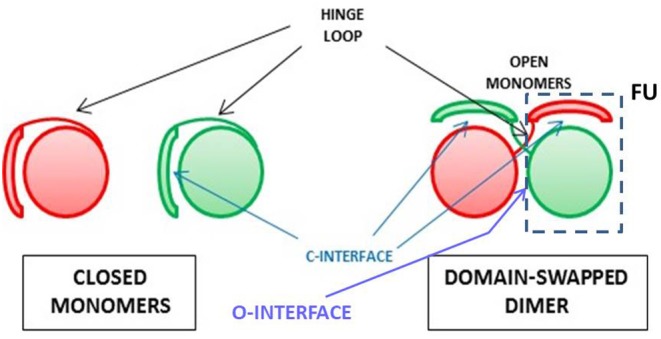
Scheme for the 3D-DS protein association mechanism. The closed interface present in the native monomer and reconstituted in the domain-swapped dimer, and the open interface(s) forming only in the dimer are indicated (241), as well as the composite functional unit (FU) (235) of the dimer inside the dashed line.

Protein oligomerization can also occur non-covalently, either as a natural or an artificial event: indeed, natively monomeric proteins can undergo dimerization or an even larger oligomerization degree. This may occur as a sort of post-translational event that can switch between active and inactive products. Many reports analyzed the features of this event involving many proteins (169, 170, 240). In the following paragraphs, we will instead focus our attention on the mechanism mainly followed by RNases to self-associate without being covalently cross-linked, i.e., the one called three dimensional domain swapping (3D-DS), whose schematic picture is shown in Figure 2.

#### RNases and the Oligomerization Through Three-Dimensional Domain-Swapping (3D-DS)

The non-covalent self-association of RNases is mainly ascribable to the 3D-DS mechanism, as reported in Table 2. Firstly defined and described by Eisenberg et al. (241, 242), and affecting many proteins (235, 243, 244), this mechanism has been deeply analyzed also by many other scientists (241, 244–247). 3D-DS partially violates the Anfinsen dogma which states that the AA sequence induces a protein to find a unique folding (10): in fact, the flexible loops of a protein (in particular, for RNase A, residues 16–22 and 112–115) can instead adopt variable conformations, thus occupying more than one energy minimum (241). This allows the domains linked to the protein flexible parts to adopt different orientations and to undergo a reciprocal exchange of a protomer domain with the equivalent domain of an adjacent subunit. Therefore, a non-covalent dimer, or even larger oligomers, if more than a single flexible loop is present, can be formed (235, 243). The domain detached from the native monomer can reconstitute the native protomer contacts in each composite, functional unit (FU) of the oligomer (Figure 2) (235). The novel FU overlaps the native monomer, except for an additional interface(s), whose conformation depend(s) to each particular protein, and that is/are absent in the native monomer (241). The domains involved in 3D-DS are often the protein N- or C-termini, or both, as it is for some RNases (235, 243). Oligomerization is often accompanied in RNases with an increase of their enzymatic and biological activities, or, moreover, with new properties absent in the native monomer. Notably, the 3D-DS mechanism is shared by many other proteins, like for example cytochrome C (248), and above all by several amyloidogenic proteins, such as human prion protein, cystatin-C or also β_2_-microglobulin, all self-associating through 3D-DS (249–252). Except for ECP/RNase 3 (46), all known RNases are unable to undergo amyloidosis, although RNase A displays many amyloid-prone segments. However, the presence of 3D-DS was not proven in ECP (253). Indeed, it is worth noting that residue(s) insertion(s) was incurred by the RNase A loops, allowing the 3D-DS to occur make it capable of forming amyloid-like domain-swapped fibrils (254, 255).

**Table 2:** List of the most important secreted RNases that spontaneously oligomerize.

**Secreted RNase**	**Oligomers**	3D-DS^*^ mechanism	**Cross-linked oligomers**	**Cytotoxic activity**
RNase A	Dimers/Trimers/Tetramers up to Tetradecamers traces	Y: N + C-swap	Native with bifunctional linkers Mutants with S-S bonds	Oligomers: debated
BS-RNase (dimeric)	Dimer Tetramers/Hexamers/Octamers	Y: N + C swap	Natural S-S bonds	Yes
RNase 1	Dimers	Y: N-swap in mutants	Mutants with S-S bonds (HHP/HHP2-RNase 1)	Oligomers: Yes
ANG	Not detected	Not detected	Only Immuno-derivatives	Oligomers: Yes
ONC	Dimer	Y: N-swap	Only in Immunoderivatives	Monomer and Dimer: Yes
Barnase	Trimer	Y: N-swap	Immunoderivative	Yes
Binase (dimeric)	Only the native dimer	Not detected.	Not detected	Yes
RNase T1	Dimer mutant	Y	Immunoderivative	Oligomeric: Yes

* *3D-DS, three-dimensional domain swapping*.

The non-covalent aggregation has been known to occur in RNase A and BS-RNase since the 60s (256–258), affecting the catalytic activity against RNA substrates. The 3D-DS involvement had already been hypothesized in 1962 by Moore et al. on the basis of the characterization of RNase S deriving from RNase A limited proteolysis that cleaves the N-terminal domain (residues 1–20) from the protein core (259, 260). RNase A aggregation was therefore already ascribed to a reciprocal exchange of the protein N-termini consequent to their detachment from the protein core (256). The actual presence of 3D-DS in RNases was then detected for BS-RNase only in 1993 by Mazzarella et al., while for RNase A in 1998 by Liu et al. (19, 233).

In the next paragraphs we list and discuss the main features of the domain-swapped dimers and oligomers of various pancreatic-type RNases, above all RNase A, BS-RNase and RNase 1, and recently also ONC, to highlight the related biological consequences. We start with RNase A, but in each paragraph comparisons within different RNase variants will be present.

#### RNase A

**Figure 3:**
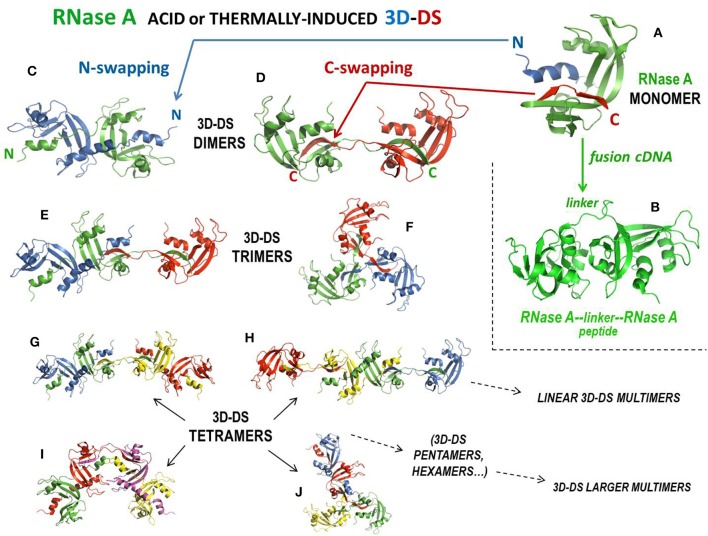
Structures of RNase A, of its tandem dimer, and of its domain-swapped oligomers. **(A)** RNase A; **(B)** covalent tandem dimer (232); **(C)** crystal structure of the N-swapped dimer, N_D_ (pdb 1A2W) (233); **(D)** crystal structure of the C-swapped dimer, C_D_ (pdb 1F0V) (234); **(E)** N + C-swapped trimer model, NC_T_ (197, 235, 236); **(F)** crystal structure of the totally C-swapped cyclic trimer, C_T_ (pdb 1JS0) (236); **(G)** N + C + N-tetramer linear model (197, 235); **(H)** C + N + C-tetramer linear model (197, 235), **(I)** N + C + N-tetramer bent model (237); **(J)** N + C + C + C-tetramer model (235).

Natively monomeric, 13.7 kDa RNase A (Figure 3A) can non-covalently self-associate by interacting with the substrate (261), but it can also oligomerize upon being subjected to lyophilization from 40% aqueous acetic acid solutions (256) or to thermal incubations in different solvents at very high protein concentration (262). Following these two protocols, RNase A produces domain-swapped dimers, trimers, and larger oligomers (115, 198, 235, 237). Oligomerization occurs through the aforementioned 3D-DS mechanism involving both N- and/or C-terminal domains of the protein (115, 235), thus producing different relative amounts of N- or C-swapped oligomers as a function of the particular protocol applied (256). Oligomers can be separated with cation-exchange chromatography, being the C-swapped species endowed with a higher basic charge exposure than the N-swapped ones (263). NMR showed that RNase A is chiefly denatured except for disulphides in 40% acetic acid, while it refolds properly, with the exception of the 3D-DS-inducing flexible loops only when, after lyophilization, it is redissolved in a “benign” buffer, such as phosphate (264). This permits the formation of many different RNase A oligomers, up to traces of tetradecamers (265). The structures of the RNase A N- and C-termini-swapped dimers (called N-dimer or N_D_ and C-dimer or C_D_, respectively), and of a cyclic C-swapped trimer, C_T_, have been solved (Figures 3C,D,F) (233, 234, 236). Additionally, models based on experimental data have been built for a N + C-swapped-trimer, i.e., displaying the swapping of both enzyme termini (NC_T_, Figure 3E), and for many other N + C-swapped tetramers (Figures 3G–J) or larger multimers (197, 235, 237). The most abundant species detectable is certainly the C-dimer that approaches 20% yield, while its amount deriving from thermal incubations is lower (197, 262). DVS or DFDNB (Figures 1E,F) crosslinking analysis confirmed that the protein self-associates through 3D-DS involving both RNase A N- and/or C-termini in all the oligomers, up to hexamers, analyzed (197, 198). The structural determinants governing RNase A 3D-DS oligomerization have been deeply investigated (266). In detail, the polarity of both RNase A N- and C-termini affects both the swapping propensity and the oligomers' stability (267), while either reducing conditions or deamidation events affecting many Asn residues reduce the tendency of RNase A aggregation through 3D-DS (268, 269). Importantly, the *cis* configuration of the X-Pro114 peptide bond present in the loop preceding the C-terminus of RNase A, RNase 1 or also BS-RNase hinders the tendency of the same terminus to be swapped (266, 270, 271). Consequently, harsh conditions are required to switch the mentioned bond from *cis* to *trans* and let the formation of remarkable amounts of RNase A C-dimer (262). In this context, it is quite surprising that about 20% of RNase A C-dimer was detected in the endoplasmic reticulum of pancreatic exocrine cells, although subsequently the dimer failed to be secreted (272). Also, glycosilation can hinder RNase 3D-DS self-association, as was demonstrated by the Asn34 N-glycosilated RNase A form called RNase B (16). Many reports indicate the actual reciprocal influence of the N- and C-termini in their swapping behavior (16, 267, 273, 274), in line with RNase S (259): indeed, this derivative dimerizes upon acidic lyophilization, but less than RNase A and obviously only through the swapping of its C-terminus (275).

All 3D-DS RNase A oligomers increase their enzymatic activity against ds-RNA substrates, or vs. DNA:RNA hybrids, with respect to the native monomer (258, 263): the increase is directly proportional to the size of the oligomers, and moreover, within the same type of oligomer, i.e., within N_D_ vs. C_D_, or NC_T_ vs. C_T_, etc. (see Figures 3C–J), the highest enzymatic activity is displayed by the species containing more C-swaps than N-swaps, because the former species expose a higher basic charge density than the latter ones (263). The antitumor activity of the oligomers has been measured too, obtaining controversial results: a relevant activity always increasing with the basic charge density of the oligomers was displayed against leukemia cells *in vitro* and *in vivo* when human melanoma cells were transplanted in nude mice (116, 168). Moreover, in similar *in vivo* experiments, this antitumor activity slightly increased when RNase A oligomers were conjugated with PEG (187). In those cases, both free and PEG-conjugated RNase A oligomers were devoid of embryotoxic activity (168, 187). Conversely, the same RNase A oligomers were not active against two human pancreatic tumor cell lines (125). However, the actual antitumor activity of the RNase A oligomers is debated because the two RNase A dimers are known to interact with RI, although with a 1:1 stoichiometry, like the aforementioned tandem RNAse A-RNase A fusion dimer (Figure 3B) that was displayed to be cytotoxic (167, 232). It must be underlined, however, that Wang et al. recently reported that RNase A, differently from RNase 1 and ANG, can induce cell proliferation ascribable to an extracellular RNase A interaction with the epidermal growth factor receptor (EGFR), in this way counteracting cytotoxicity as well (276). Hence, from what has been reported, the evaluation of the actual antitumor potential of RNase A species certainly deserves further investigation.

#### BS-RNase

**Figure 4:**
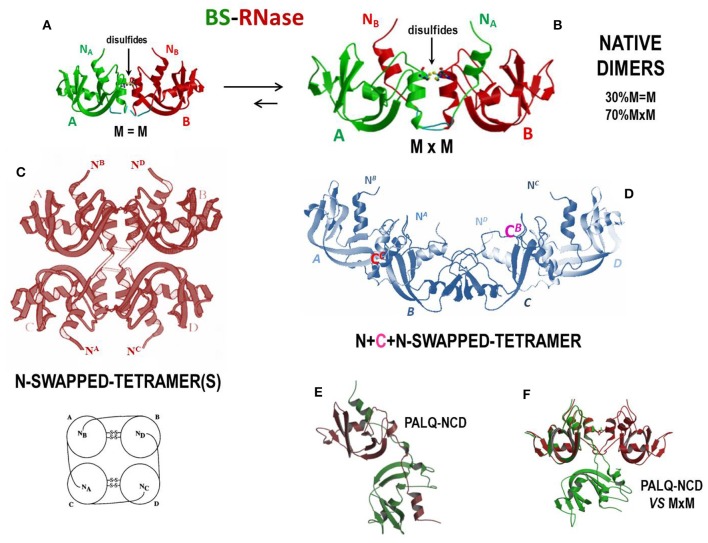
Structures of BS-RNase and of its tetrameric derivatives. **(A)** BS-RNase unswapped native dimer isoform, M=M, about 30% of the total (278, 279); **(B)** N-swapped native dimer isoform, M×M, about 70% of the total (19, 278); **(C)** totally N-swapped cyclic tetramer model plus schematic model (280); **(D)** N + C + N-swapped tetramer (199); **(E)** PALQ-BS RNase mutant non-covalent dimer and **(F)** its comparison with the N-swapped BS-RNase wild type (281).

As mentioned, BS-RNase is the unique natively homo-dimeric member of the pancreatic-type RNase superfamily because of the two antiparallel disulfides linking the Cys31 residue of one subunit and the Cys32 of the other, and vice-versa (277). These two residues are absent in other RNases. However, each BS-RNase subunit is composed of 124 AA residues and displays 82% sequence identity with RNase A (17). Interestingly, native BS-RNase is an equilibrium mixture between two isoforms (Figures 4A,B), one of which (~70%) is characterized by the swapping of its N-termini and is represented as M×M (panel B), while the other (~30%) is unswapped and named M = M (panel A) (278), given that only the two inter-chain C31-C32/C32-C31 disulfides permit the dimer formation. This M×M/M = M equilibrium has been deeply investigated (282) and partially affects the catalytic activity of the enzyme (278). The role of the inter-subunit disulfides has also been analyzed (283, 284), as well as the one of the hinge loop connecting the N-terminus with the protein core. In addition, P19 and L28, as well as R80 residues, have been shown to influence the M = M vs. M×M equilibrium (285–287).

The ribonucleolytic activity of the enzymes present in the bovine seminal fluids was firstly described by D'Alessio and Leone in 1963 (288). The responsible agent for this activity was discovered to be BS-RNase, that is definitely more basic and more active against ds-RNA adducts than RNase A (289). This is true also after monomerizing the enzyme by the reduction of the two inter-subunit disulfides (290), thus confirming that the basic charge density, and not the dimeric nature *per se*, is crucial for a RNase to be active against ds-RNA substrates. Moreover, as reported before, BS-RNase exerts a remarkable antitumor activity (162, 291, 292), together with immunosuppressive, embryotoxic and aspermatogenic effects (73, 75–77). Cytotoxicity is selectively addressed only against malignant cells, also of human origin (125, 293, 294), and is ascribable only to the N-swapped M×M isoform, while neither M = M nor the wt-monomeric derivative are active (162, 164). This feature has been ascribed to the ability of only M×M to evade cellular RI: in fact, the cytosolic reducing environment, although breaking the two inter-subunit BS-RNase disulfides, does not monomerize the M×M isoform, whose dimericity survives, although as a non-covalent dimer (NCD), for a sufficient time to exert its cytotoxic action. The unswapped M = M isoform instead undergoes monomerization upon losing the two mentioned disulfides and becomes therefore susceptible to the RI blockage (164). The crystal structures of the M = M, M×M (Figures 4A,B), and NCD isoforms of BS-RNase, as well as of the monomeric derivative, have been solved, in this way clarifying crucial structural features that govern the possibility of this enzyme to exert its biological activities (19, 279, 295, 296). Importantly, the M×M isoform does not vary its conformation upon losing its inter-subunit disulfides and forms NCD only if the P19 and L28 are conserved, because these residues allow NCD to maintain the capability to evade RI (see Figures 4E,F). The P19 and L28 mutations induce instead a dramatic variation in the reciprocal orientation of the two subunits that renders the mutated NCD variant inactive because it becomes susceptible to RI (281). However, mutants of the monomeric BS-RNase derivative designed to evade RI exerted a 30-fold higher cytotoxic activity than the wild-type dimer (292).

As well as RNase A (269, 297), BS-RNase easily undergoes Asn67-to-Asp deamidation, an event that is even quicker than for the pancreatic variant (298). However, the acquired negative charge of Asp67 does not affect its cytotoxicity. Additionally, many efforts have been performed to increase the RNase A/BS-RNase similarity to make also the pancreatic variant endowed with antitumor activity. To this end, dimerizing RNase A variants built through the introduction of the K31C/S32C mutations, in addition to others, was revealed to exert relevant antitumor action against malignant fibroblasts (299, 300), while the cytotoxicity of an engineered RNase A/BS-RNase hybrid displaying a N-swapped dimeric structure was not measured (301).

Interestingly, BS-RNase can also be induced to self-associate upon acetic acid lyophilization. In this way, tetramers, hexamers and even larger oligomers can be formed: two different tetramers forming through the swapping only of their N-termini were hypothesized and modeled by Mazzarella et al. (Figure 4C) (280), while more recently it has been demonstrated that BS-RNase can swap also its C-termini, as RNase A does. The C-termini swapping can occur either in the native dimer, to form a N + C + N-swapped tetramer (Figure 4D) (199), or also involve the BS-RNase monomeric derivative to produce a C-swapped dimer structurally similar, but not identical, to the RNase A C_D_ (302). Importantly, BS-RNase oligomers also display enzymatic and cytotoxic activities higher than the native dimer; again, both activities increase with the size of the oligomers and, consequently, with their basic charge density (199). To this regard, many structural, enzymatic and antitumor features of RNase A and BS-RNase oligomeric species are compared and discussed in (117).

#### RNase 1 or HP-RNase

**Figure 5:**
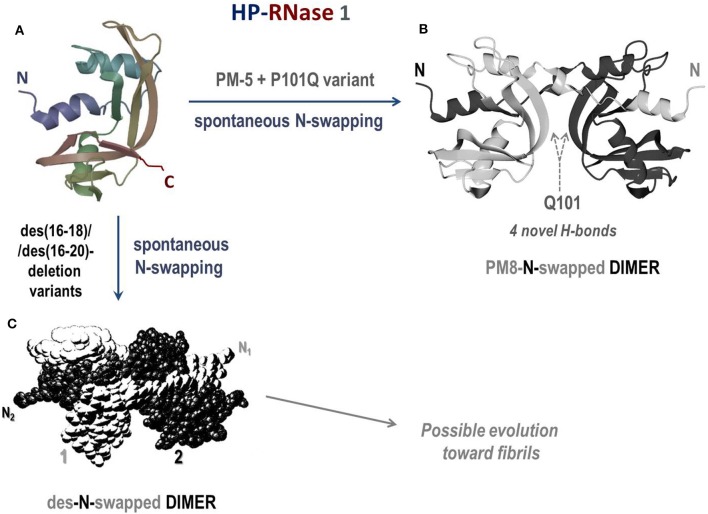
Structures of the human pancreatic RNase 1 and of the dimers of two of its mutants. **(A)** HP-RNase 1; **(B)** crystal structure of the N-swapped dimer of PM8 (PM5 + P101Q) mutant (pdb 1H8X) (303); **(C)** des-N-swapped dimer (304).

Human pancreatic (HP) RNase 1 (Figure 5A) is natively monomeric, but, being 128 AA residues long, it displays a C-terminus elongation of four residues with respect to its bovine homolog RNase A, and it is more basic than it (266, 305). This elongation does not affect RNase 1 stability and reduces only slightly its enzymatic activity (306). Moreover, this variant is definitely more active than RNase A vs. ds-RNA substrates (12), reaching a maximal value at pH 7.3. This pH is very close to the one of the blood and definitely higher than 6.5, the value under which RNase A exerts its maximal activity (12). Therefore, considering the high basicity of its native state, RNase 1 has been mutated to insert, as well as in RNase A (299), two 31 + 32 cysteine residues to make it dimerize similarly to BS-RNase: these mutants, named HHP-RNases, displayed a remarkable antitumor activity against several types of malignant cell lines, among which cells from neuroblastoma and rhabdomyosarcoma showed the highest sensibility (307). Indeed, the RNase 1 variant displaying the Cys31 + 32 couple plus the N28L/N34K/E111G mutations, and called HHP2-RNase, exerted a quite high antitumor activity against human thyroid carcinoma-derived cell lines and *in vivo* when malignant cells were transplanted into nude mice (308). Cytotoxicity was exerted by both swapped and unswapped isomers, contrarily to BS-RNase, and this result was ascribed to the stabilization effect of both the hinge loop and the Leu28 side chain in terms of maintaining the two Cys31/32 residues close to each other (309). More recently, other different Cys31/32 cytotoxic dimeric mutants have been engineered to evade RI, and they were displayed to exert a relevant cytotoxic activity as well (310).

Other RNase 1 mutants able to swap their N-termini have been produced: five-residue mutant (PM5) that makes the RNase 1 N-terminal edge identical to BS-RNase displayed a higher stability than the wild type (311). Moreover, an additional P101Q mutation introduced in this variant allowed RNase 1 to spontaneously dimerize through 3D-DS thanks to the formation of four novel inter-subunits H-bonds (Figure 5B). This additional mutation permits also stacking interactions between the β-sheets of each protomer to be gained, similarly to what occurs in the RNase A N_D_ (303). Hence, this RNase 1 N-swapped dimer, called PM8, combines the structural determinants of BS-RNase (five mutations in the N-terminus, PM5) with the crucial role of Gln101 detected in the RNase A N-dimer (233, 312). However, the structure of this dimer (303) is quite different from both BS-RNase M×M isoform because the Cys31-32 disulfides are absent (19, 233). Again, deletions suffered by the loop connecting the N-terminus to the protein core (des(16-18) or des(16-20)HP-RNase) induced the spontaneous formation of stable dimers forming through the domain swapping of the N-termini (Figure 5C), as confirmed by the DVS cross-linking (304). Interestingly, the des(16-20) HP-RNase mutant was recently discovered to form supramolecular structures resembling amyloid-like rod-shaped fibrils (313). The structural features of many dimerizing RNase 1 variants have been deeply analyzed (314), while their antitumor activity has not been investigated so far.

#### Onconase (ONC)

**Figure 6:**
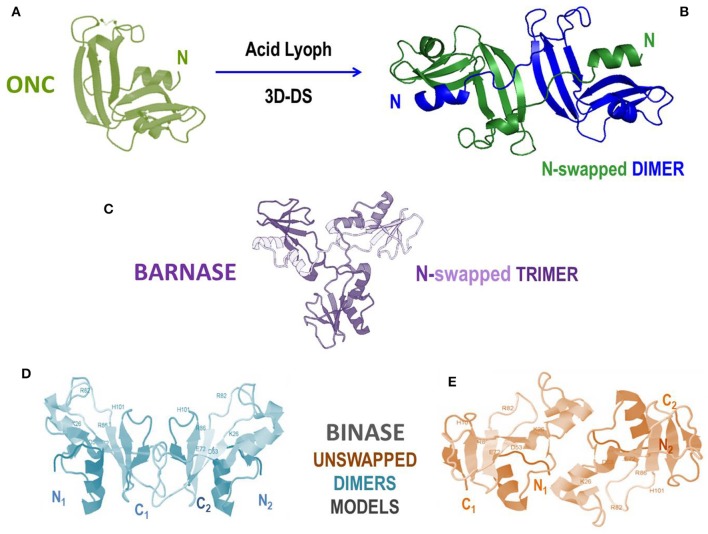
Structures and models of non-mammalian RNases and of their oligomers. **(A)** Amphibian onconase (ONC); **(B)** N-swapped ONC dimer model (200); **(C)** crystal structure of the N-swapped cyclic trimer of bacterial barnase (pdb 1YVS) (315); **(D,E)** two alternative models for the bacterial natively dimeric unswapped binase, stabilized by electrostatic interactions at the subunits' interface (90).

Extracted from the *Rana pipiens* oocytes, ONC is the unique pancreatic-type RNase known to be remarkably cytotoxic in its very stable native monomeric form (Figure 6A) together with the less investigated, but also amphibian, amphinase variant (27). Although being the smallest [104 AA, MW 11.8 kDa (28)] pancreatic-type RNase, and being also not highly catalytically active, ONC displays the same catalytic triad (here H10/K31/H97) and catalytic subsites similar to the RNase A ones as well (316). Furthermore, beyond its antitumor effect, ONC displays a prominent antiviral action by upregulating factors that inhibit viral genome replication (317). ONC can penetrate cancer cells because of its high basicity and favorable interaction with the sialic acids moieties present on the malignant cell membranes (318). Then, the crucial step is that ONC evades RI because it lacks the key residues necessary to form a tight complex with the inhibitor (152). ONC can attack tRNAs, as well as other substrates, such as miRNAs, to exert its cytotoxic action (319–321). Thus, at first glance, it would seem unnecessary to produce ONC supramolecular structures to design anticancer therapies (322); in fact, many positive results have been registered with monomeric wt-ONC both *in vitro* and *in vivo* against several still incurable tumors, like pleural mesothelioma, human lung, glioma, pancreas and melanoma (27, 323–326). However, the although reversible renal toxicity of ONC discovered upon clinical trials (188) partially cooled down the initial enthusiasms. This somehow alerts to find a way to enlarge the dimensions of ONC moieties to make their filtration at the glomerular barrier more difficult, and increasing its circulating half-life at the same time. Consequently, many fusion immune-ONC derivatives have been successfully built, as we reported in the previous paragraphs and in Table 1 (218). Then, notwithstanding its remarkable stability (T_M_ ~ 90°C), ONC has been recently discovered to form a N-swapped dimer upon lyophilization from acetic acid (Figure 6B) (200). Notably, this dimer displayed to be more cytotoxic against pancreatic cancer cells than the corresponding native ONC monomer (200). Unfortunately, ONC can swap only its N-terminus, being the C-terminus locked by the disulfide involving the Cys87 and Cys104 terminal residues. The impossibility to swap more than one domain definitely reduces the protein self-association propensity in general (243), and this is certainly the case of ONC. Moreover, some ONC variants built to unlock its C-terminus were found to be less stable than the native enzyme (327, 328). This suggests a negative influence of the free ONC C-terminus in the stabilization of the N-swapped dimer, confirming also for ONC the reciprocal influence of the N- and C-termini in the 3D-DS event involving RNases (200). Consequently, the unique way known to date to obtain large ONC homo-oligomers useful to escape renal filtration is the use of the aforementioned trifunctional maleimide to produce covalent derivatives (see Figure 1D) (172).

However, and finally, we mention a very recent study that exploited both ONC and RNase A features to build a chimera able to remarkably augment the tendency of the latter mammalian enzyme to undergo 3D-DS oligomerization. This was obtained by substituting in RNase A the 112–115 C-terminal loop, comprising the Pro114 residue, with the shorter loop present in ONC and devoid of this proline key-residue (271).

#### Microbial Barnase and Binase

Barnase and binase, both RNase variants of about 12 kDa, belong to the RNase N1/T1 microbial superfamily. Importantly, we firstly recall that in the past a mutant of fungal RNase T1 was shown to acquire activity against ds-RNA upon dimerization through 3D-DS (see Table 2) (329). Conversely, the wt-RNase T1 is not cytotoxic, being unable to enter the cells if it is not pre-incorporated into a HVJ cell-penetrating envelope vector (330). Then, barnase was in turn discovered to form a cyclic domain-swapped “flower-like” trimeric adduct (Figure 6C) (315). Furthermore, barnase interaction with its inhibitor barstar has been recently discovered to induce the lysis of staphylococcal bacteria (112). We underline again that barnase was derivatized, either as a monomer or as a dimer, also with immuno-peptides to form Immuno-RNase derivatives that were cytotoxic against breast and ovarian cancers (87–89). Binase, instead, is a variant known to display a remarkable toxicity toward many tumor cells (83, 91), and is also accompanied with a relevant antiviral activity (92, 93, 331). Binase has been subsequently found to exist as a natural dimer (90, 332), and the presence of the swapping of the N-terminal domain has been strongly suggested. However, two alternative structural models did not confirm the actual presence of 3D-DS: in fact, two structures showing electrostatic interactions have been proposed as being able to stabilize the interface between the two monomeric subunits of the native binase dimer (Figures 6D,E) (90). Interestingly, the variant balifase has also been characterized, apparently displaying a lower cytotoxicity than binase (84). Hence, further investigations on the structural determinants governing the important antitumor activity of these microbial RNases certainly deserve be performed to design future efficacious anti-cancer, as well as antimicrobial but possibly not immunogenic, RNase derivatives.

## Conclusions and Future Perspectives

Several aspects of the catalytic, immunomodulatory and antitumor properties displayed by many RNases and by their oligomeric derivatives have been described in this review. Some results useful for therapy against still incurable cancers have been underlined or suggested as well. Although promising results have been already obtained with some RNases, and especially with ONC, improvements are necessary in terms of potentiating cytotoxicity and contemporarily attenuating or deleting undesired side-effects. To this end, RNases homo- or hetero-dimerization, or more extensive oligomerization, might certainly represent an efficacious strategy to be sharply tuned.

The hetero-crosslinked immuno-RNases or the homo or hetero fusion-proteins have certainly offered promising results that could be hopefully transferred toward clinical use in the next future. However, the spontaneous or artificially induced non-covalent RNase self-association may also represent a fruitful pathway to produce active RNase derivatives.

Hence, the oligomerization tendency of, and the products formed by RNase A, BS-RNase, RNase 1 and ONC may suggest a smart future approach to build complex chimera products. From what has been reported, it emerges that the determinants driving a RNase to dimerize or oligomerize in a way that can make it cytotoxic depend on several features, comprising 3D-DS propensity, stability, basic nature of key-residues and geometry of the supramolecular adduct(s) formed. These features can affect crucial steps correlated with cytotoxicity, such as the interaction with the cell membrane, ability to enter the cytosol or to evade RI.

Therefore, and finally, the RNase oligomerization strategy should drive toward products accompanied with relevant antitumor activity but devoid of undesired side-effects. Moreover, the RNase derivatives should be characterized by a satisfactory half-life in the circulatory system, to reach more easily the tumor place, thus allowing successful applications in therapy.

## Author Contributions

Both authors contributed to the manuscript: GG more about the structural and functional features of RNase oligomers, MM at higher extent about the biological aspects related to the activities of the RNase oligomers. GG built the figures and checked the overall english quality. MM critically helped to make the manuscript as much coherent and homogeneous as possible.

### Conflict of Interest

The authors declare that the research was conducted in the absence of any commercial or financial relationships that could be construed as a potential conflict of interest.
